# Endoscopic Findings of Early Stage Vocal Fold Cancer

**DOI:** 10.1155/DTE.1.185

**Published:** 1995

**Authors:** Peter Kitzing

**Affiliations:** 1 Department of Phoniatrics University ENT-Clinic Malmö General Hospital Malmö Sweden; 2 Foniatriska avd. Öronkliniken Malmö S-21401 Sweden

## Abstract

To describe early cancerous and precancerous lesions of the laryngeal vocal folds as well as of the most common differential diagnoses, based on a series of microlaryngoscopic photographs. Some introductory remarks about terminology and the classification of epithelial lesions of the vocal folds are included. The paper ends with some comments as to the management of epithelial thickenings (or leukoplakias) of the vocal folds. Malignancy should be suspected as long as it has not been ruled out by histologic diagnosis on adequate biopsies, which is the only way to correctly evaluate the character of such lesions. Precancerous lesions should be controlled by regular follow up examinations as carefully as invasive carcinomas (posttreatment), because there is a high tendency for recurrences or for later development of malignancy in these cases.


*Diagnostic and Therapeutic Endoscopy,* 1995, Vol. 1, pp. 185–194
Reprints available directly from the publisher
Photocopying permitted by license only

© 1995 Harwood Academic Publishers GmbH
Printed in Singapore

### Endoscopic Findings of Early Stage Vocal Fold Cancer

PETER KITZING

*Department of Phoniatrics, University ENT-Clinic, Malmö General Hospital, Malmö/Sweden*

*(Received April 5, 1994; in final form November 28, 1994)*

To describe early cancerous and precancerous lesions of the laryngeal vocal folds as well as of the most common differential diagnoses, based on a series of microlaryngoscopic photographs. Some introductory remarks about terminology and the classification of epithelial lesions of the vocal folds are included. The paper ends with some comments as to the management of epithelial thickenings (or leukoplakias) of the vocal folds. Malignancy should be suspected as long as it has not been ruled out by histologic diagnosis on adequate biopsies, which is the only way to correctly evaluate the character of such lesions. Precancerous lesions should be controlled by regular follow up examinations as carefully as invasive carcinomas (posttreatment), because there is a high tendency for recurrences or for later development of malignancy in these cases.

**KEY WORDS:** endolaryngeal microsurgery, microlaryngoscopy, vocal fold cancer, vocal fold epithelial dysplasia, laryngeal intraepithelial neoplasia

### INTRODUCTION

Cancer of the larynx accounts for almost 2% of all cancer diagnosed in males but for less than 0.5% in females. The male versus female ratio varies among countries, the majority falling into the range of 6 to 10 males to 1 female. During the recent decades, the incidence tends to increase in females, whereas it seems rather stable in males. The age range varies from 50 to 80 years in the majority of cases, but there are exceptional reports of cases under the age of 20.

Three regions of cancer location in the larynx are generally recognized: supraglottic, glottic (vocal fold), and subglottic. The proportion of subglottic cancers is mostly reported to be low. The ratio of glottic to supraglottic varies among countries as well as between ethnic and social groups. The reason is not clearly understood so far. In Sweden, as in the majority of North European countries with the exception of Finland, glottic carcinoma is most common (1,2).

The survival rate for early (stage Ia and Ib) vocal fold (glottic) carcinomas is in the magnitude of 90% or more, whereas there is a drastic drop to about 70% for stage II and down to about 50% for stage III and IV cases. At stage III and IV, healing usually can be achieved only at the price of mutilating surgery, *i.e.*, partial or total laryngectomy, with loss of the natural voice and other impediments of the quality of living as an obvious consequence. On the other hand, in early stages, radiotherapy is sufficient to cure the cancer in most cases, thereby preserving a natural breathing and voice function. Therefore, early diagnosis is of great importance. Not only the specialized laryngologist but every endoscopist of the airways should be aware of the findings of early vocal fold cancer and of the epithelial lesions that are its precursors or represent a differential diagnosis.

### TERMINOLOGY

There tends to be some confusion in the terminology of pathological findings on the vocal folds. One may see whitish thickenings of the vocal fold epithelium be described as chronic laryngitis, polyp, leukolakia, keratosis, hyperkeratosis, papilloma, etc. The correct designation for most of such findings will be leukoplakia, meaning just a white patch in Greek. This is merely a description of the clinical finding, covering the fact that a differentiated diagnosis can be established only by microscopic evaluation of biopsies. As will be seen from the photographs

Address for correspondence: P. Kitzing, M.D., Foniatriska avd., Öronkliniken, S-21401 Malmö/Sweden

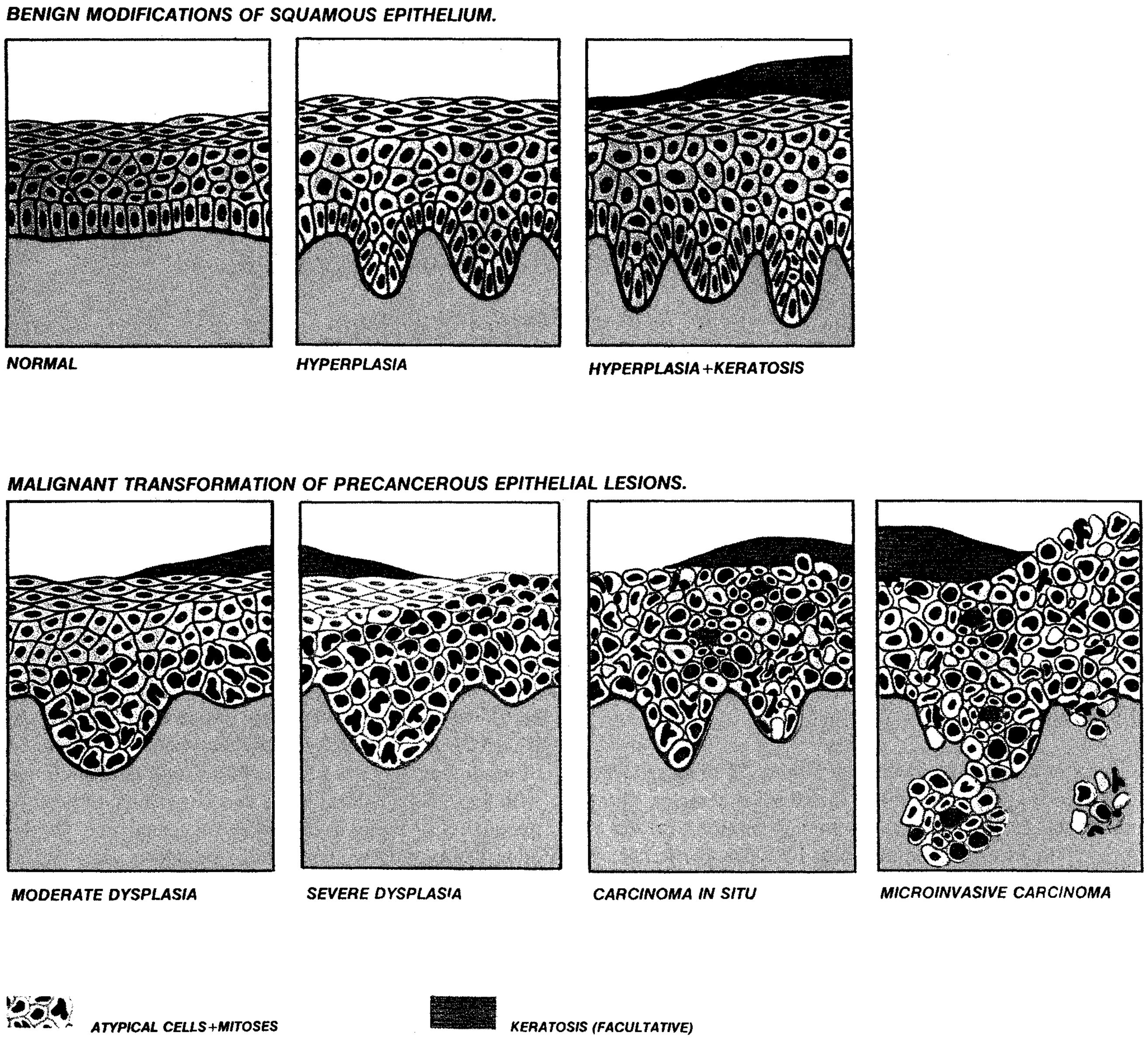

**Figure 1** Schematic representation of the histology of vocal fold epithelial changes (from Lehmann *et al.*, (3) by permission).

presented here very similar clinical findings may represent entirely different histopathological diagnoses, and a certain pathological diagnosis may present itself as a diversity of clinical appearances in different cases.

Histologically, by far most of the malignant neoplasms of the larynx (above 95%) are squamous cell carcinomas. They may arise either from stratified squamous epithelium such as on the free edges of the vocal folds, or from columnar (ciliated) epithelium, which has undergone squamous metaplasia.

### CLASSIFICATION OF EPITHELIAL LESIONS

The majority of suspect epithelial lesions of the vocal folds are histologically diagnosed as invasive carcinomas already at their first presentation (3–5). However, most authors recognize different changes of the epithelium, some of which are interpreted as precursors of carcinoma, so called precancerous lesions, even if it still seems unclear if their development into a carcinoma is obligatory. On the contrary, early epithelial changes may be reversible, and invasive carcinoma may very well emanate from normal squamous epithelium. On the other hand, the epithelium close to an invasive carcinoma very often shows precancerous alterations (Fig. 8), or a cancer of one vocal fold may be accompanied by precancerous epithelial lesions on the other side (Fig. 4).

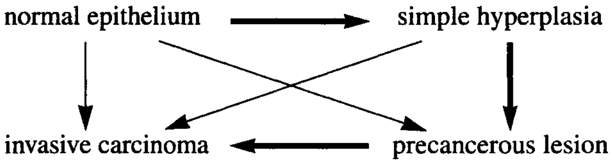

Kleinsasser (6) conceptualized the development of invasive carcinoma of the vocal folds by the following diagram, in which he questioned a development according to the thin arrows. He also proposed a widely accepted classification (Kleinsasser 1963) into (*a*) simple epithelial hyperplasia without atypias; (*b*) epithelial hyperplasia with atypia; and (*c*) precancerous epithelium including carcinoma in situ.

As the dysplasias of the epithelium nowadays are considered to be the morphological manifestations of a neoplastic process, the more modern concept of "laryngeal intraepithelial neoplasia (LIN)" is used in a recent classification by Friedman *et al.* (8) (cf. also Fig. 1):

keratosis, hyperplasia
dysplasia: low grade (LIN I)
dysplasia: moderate (LIN II)
dysplasia: high grade and carcinoma in situ (LIN III)
microinvasive carcinoma

### PRECANCEROUS AND CANCEROUS EPITHELIAL LESIONS OF THE VOCAL FOLDS. ENDOSCOPIC APPEARANCE AND DIFFERENTIAL DIAGNOSIS

Epithelial thickening often seems to be caused by long standing irritation, e.g., from tobacco smoking or from chronic inflammation. In chronic laryngitis, thickened and inspissated mucus may be glued to the mucosa, and even at the endoscopic examination it may be difficult to distinguish from leukoplakia caused by epithelial thickening and keratosis (Fig. 2).

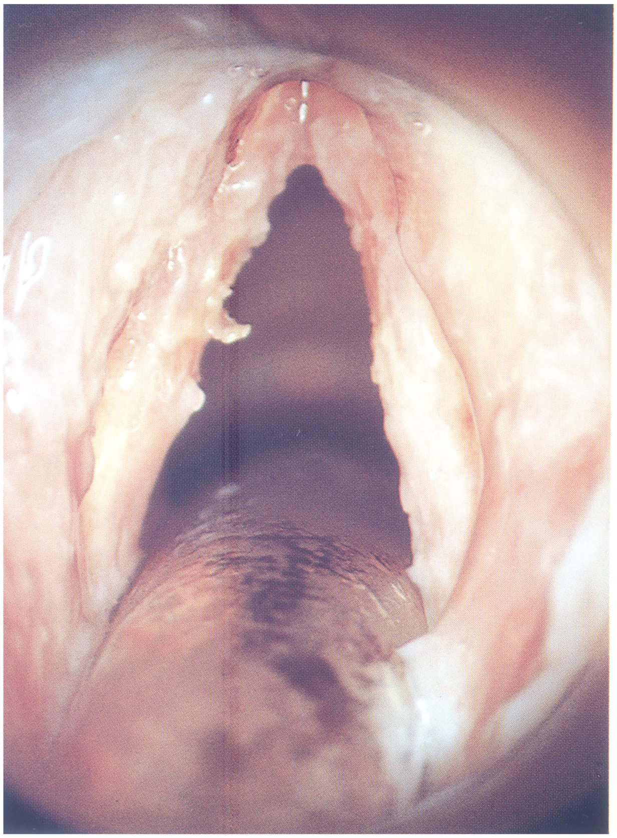

**Figure 2** Chronic laryngitis; woman, 71 years old. Slightly thickened but entirely pliable mucous membranes covered with imspissated mucus, difficult to distinguish from leukoplakia.

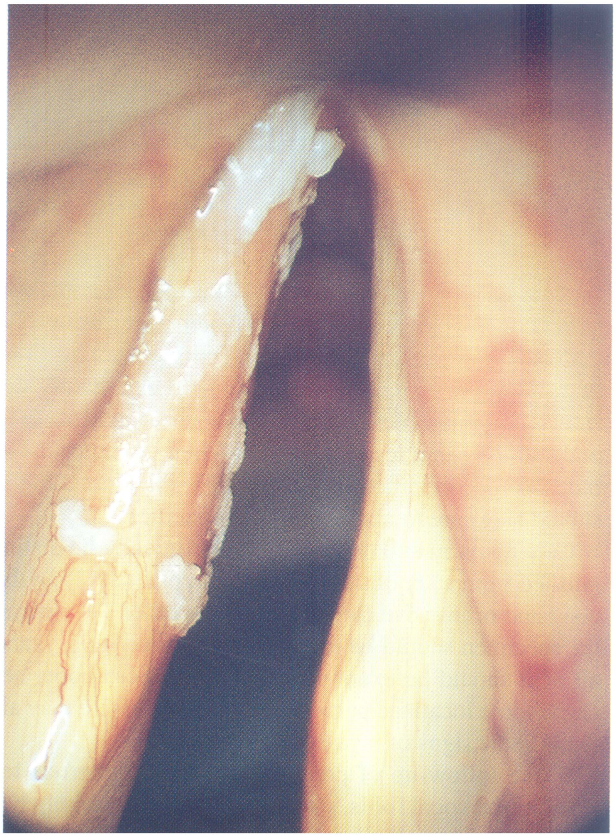

**Figure 3** Leukoplakia of left vocal fold; man, 75 years old. Well demarcated borders in some parts, blurred opacities in others. Histology: Keratosis with moderate dysplasia (LIN II).

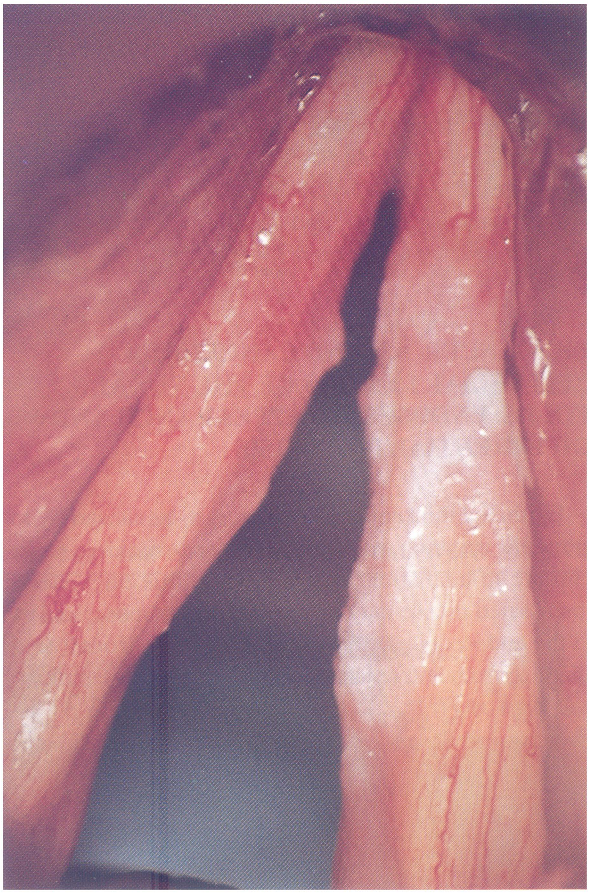

**Figure 4** Carcinoma in situ of right vocal fold; man, 57 years old. Opaque leukoplakia covering tortuous and widened vessels. Reddish irritation and epithelial thickening (LIN II) of left vocal fold.

Depending on the degree of keratosis, leukoplakias caused by dysplasias may present themselves as opaque thickenings of the epithelium with continuous transitions into the normal mucosa or they may show sharply demarcated borders. Both types of boundaries may occur in one and the same lesion (Fig. 3). When taking excision biopsies (decortication or "stripping"), the laryngologist should take care to also dissect the opacities.

Carcinoma in situ (Fig. 4) cannot clinically be distinguished from a keratosis with slight atypia (LIN I) on the one hand or invasive carcinoma on the other. As always, the specimen should be taken as an excision biopsy, and all of it should be examined under the microscope, because some localized parts of a carcinoma in situ may already show signs of invasive growth.

In the follow-up of precancerous and cancerous lesions of the vocal folds, stroboscopy may be of help because the normal vibrations of the vocal folds are arrested by invasive lesions (9) The method may be very sensitive, as is illustrated in Figure 6, where an invasive carcinoma of only a few square millimeters size is shown, with localized vibratory standstill at stroboscopy at a follow-up 4 years after an earlier simple keratosis (Fig. 5).

Early invasive exophytic carcinomas often have a typical granulomatous appearance, which raises a strong suspicion of malignancy already at the clinical examination and endoscopy (Figs. 7 and 8). Sometimes, however, they may mimic benign lesions such as papillomas (Figs. 9 and 10) or polyps (Figs. 11 and 12).

The experienced endoscopist should be familiar with the typical appearance of the vocal folds after radiotherapy. Initially, irradiation often causes a generalized edema, but after several months this is usually superseded by a more atrophic appearance of the larynx, the color of the vocal folds being white as chalk and with a pattern of crimson red tortuous vessels (Fig. 13).

Mostly the correct diagnosis of vocal fold epithelial lesions is impossible only from the endoscopic appearance. So, it must be admitted that the diagnosis of laryngeal tuberculosis (Fig. 14) came as a total surprise to the present

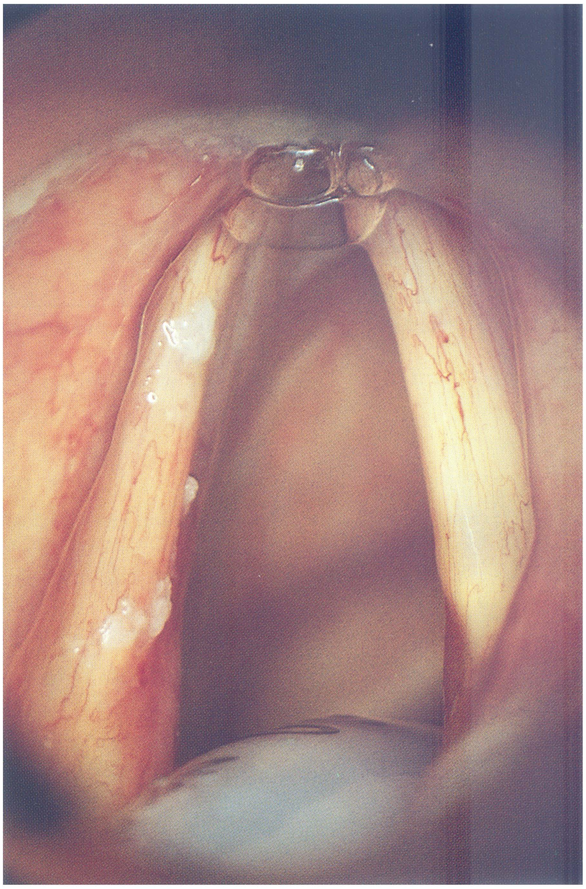

**Figure 5** Patchy epithelial thickening of left vocal fold; man, 65 years old. Histology: keratosis—hyperplasia, no atypia.

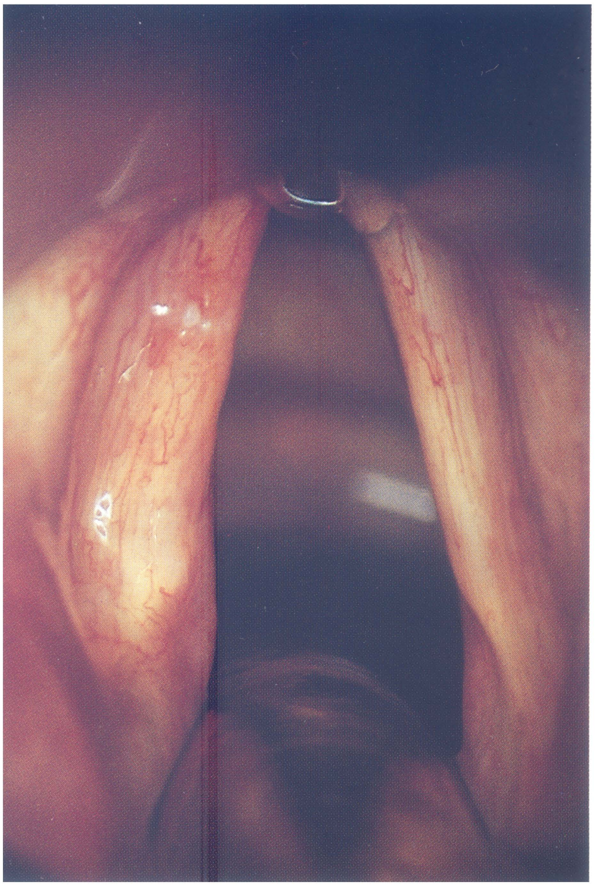

**Figure 6** Same subject as in Fig. 5, 4 years later. Small whitish lesion (to the right of light reflex) on anterior third of left vocal fold and surrounded by tortuous vessels. Histology: invasive squamous cell carcinoma.

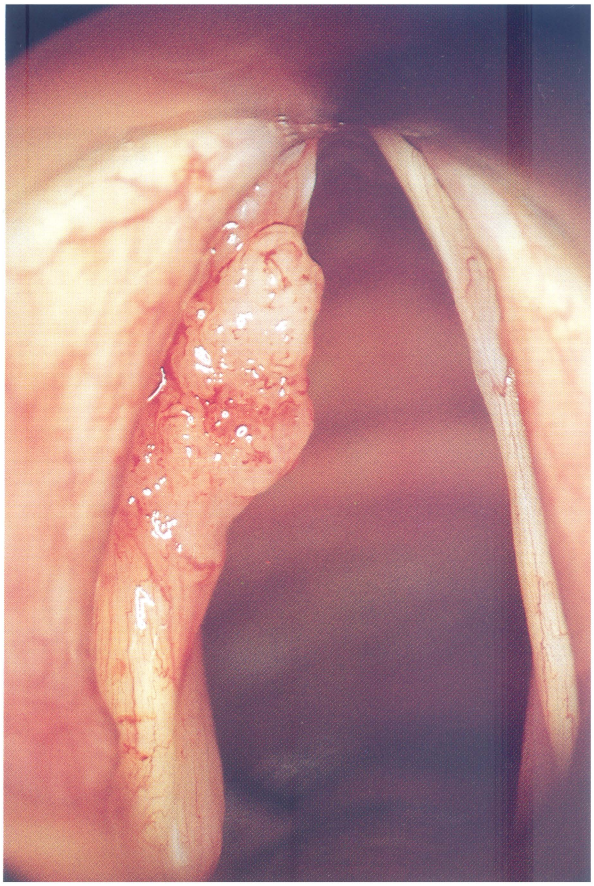

**Figure 7** Exophytic granulomatous invasive carcinoma of left vocal fold, male, 67.

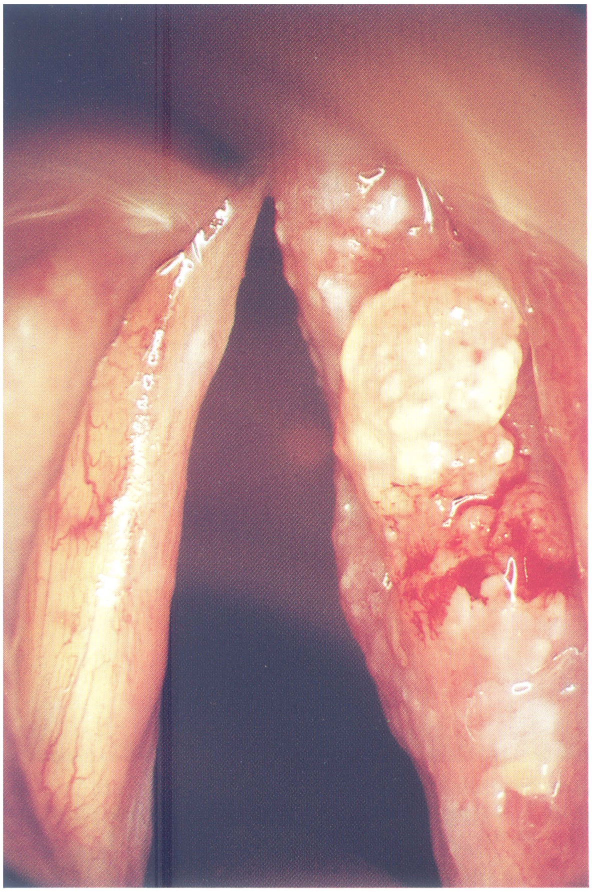

**Figure 8** Exophytic granulomatous invasive carcinoma of right vocal fold; man, 53 years old. Notice areas of carcinoma in situ anterior and posterior to the exophytic lesion.

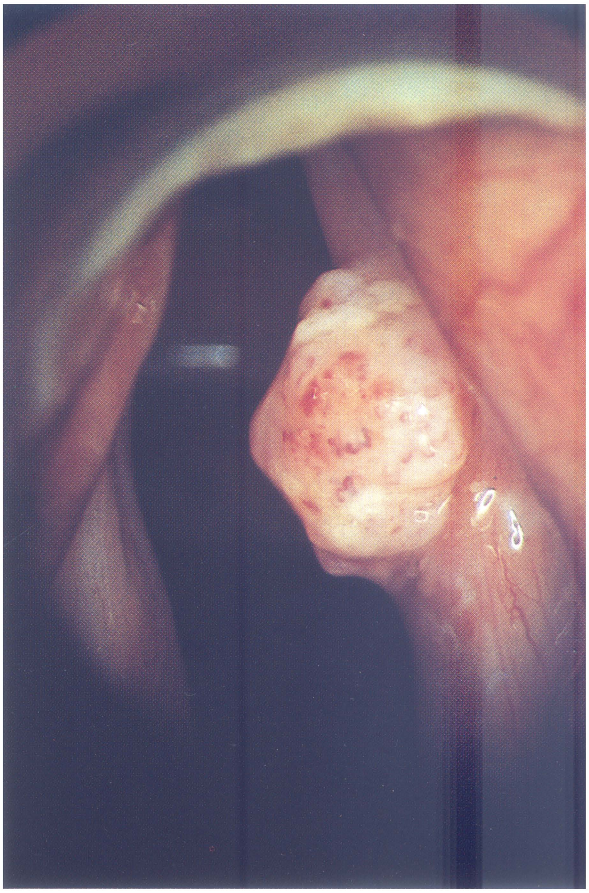

**Figure 9** Exophytic well demarcated granulomatous invasive carcinoma of right vocal fold; man, 84 years old. Cf. Fig. 15.

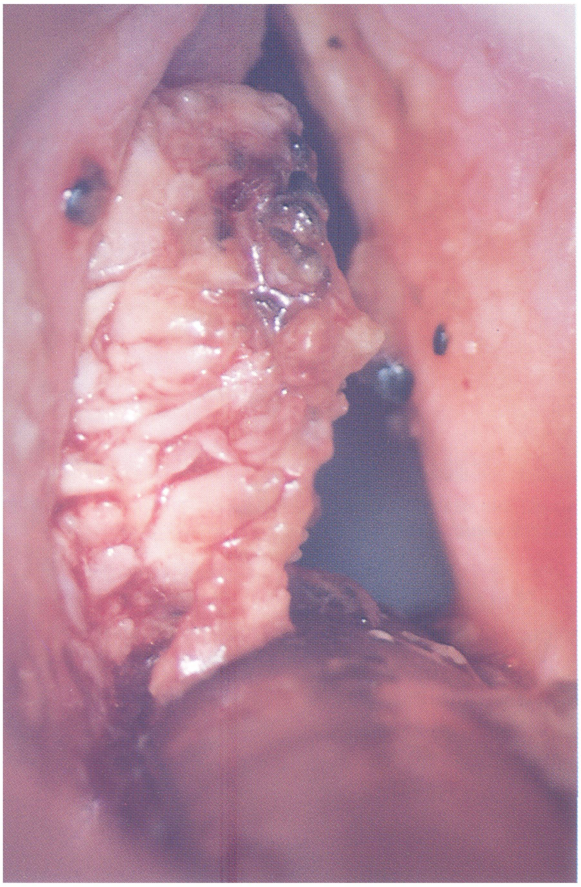

**Figure 10** Papillomatous lobated invasive carcinoma of left vocal fold with small hematoma in its anterior (upper) part, man, 58 years old. Histology: well differentiated invasive squamous cell carcinoma. Notice gross appearance not differing from benign juvenile papilloma.

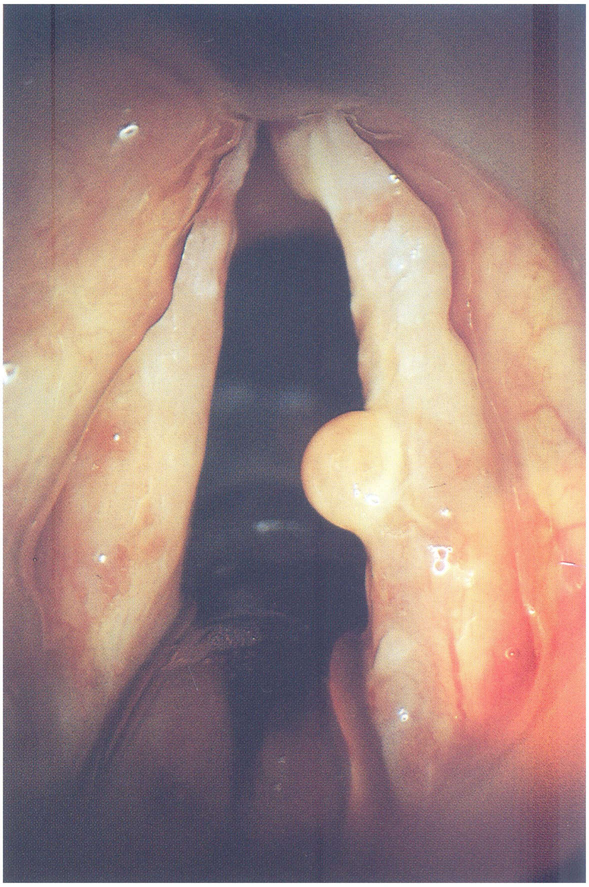

**Figure 11** Polypous invasive carcinoma; man, 74 years old. Identical appearance as benign polyp.

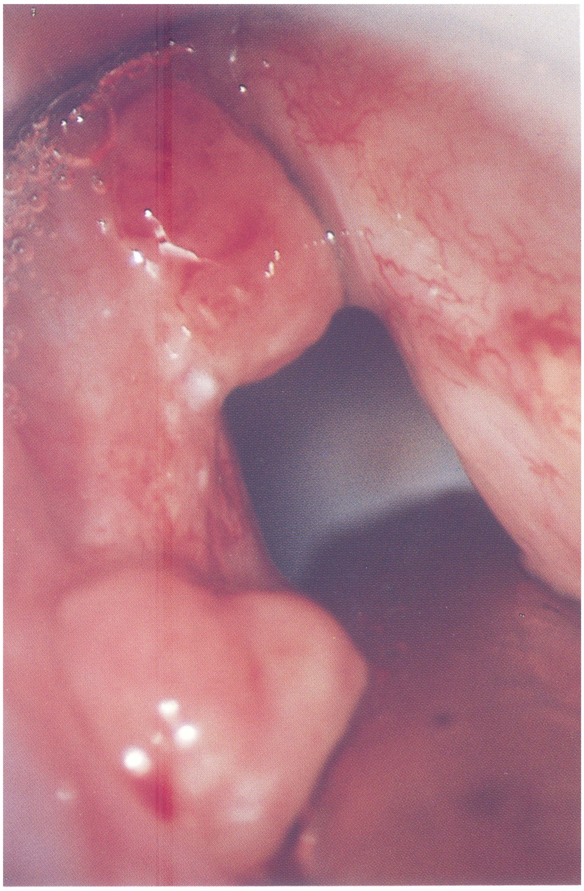

**Figure 12** Polypous protrusions on anterior and posterior thirds of left vocal fold, representing recurrence of invasive carcinoma after radiotherapy 2 years earlier; man, 63 years old.

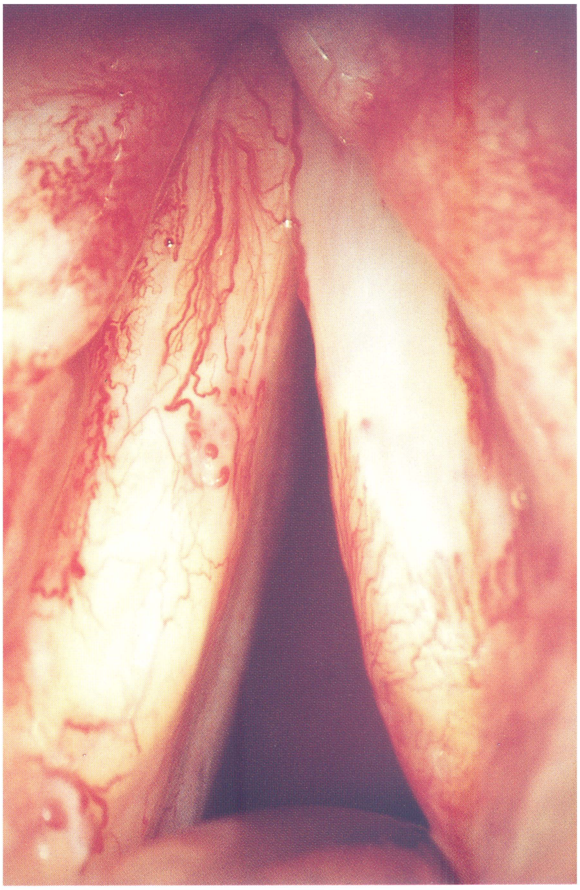

**Figure 13** Typical appearance of vocal folds after radiotherapy; man, 52 years old. Irradiation carried out 2 years earlier. Notice whitish colour of vocal folds with tortuous vessels.

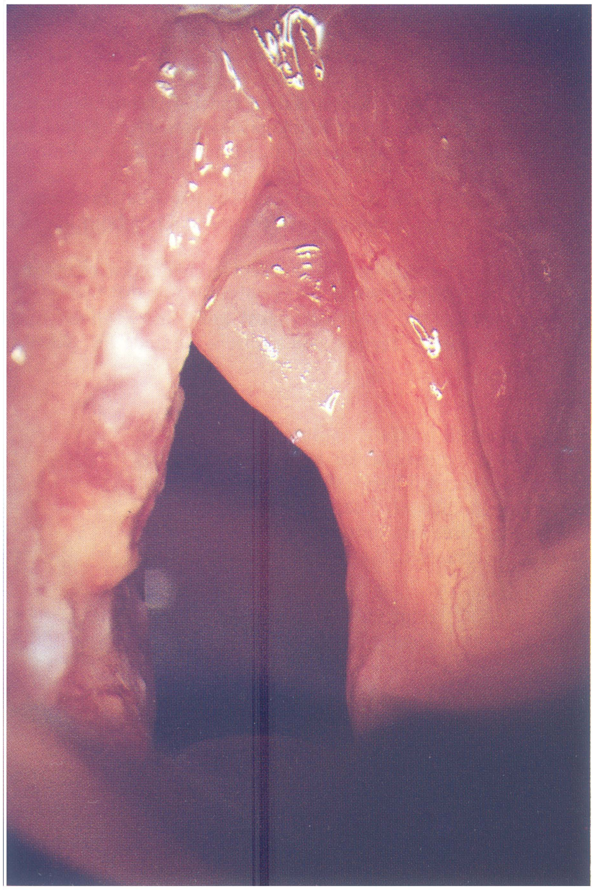

**Figure 14** Tuberculosis of left vocal fold; man, 36 years old. The right vocal fold shows polypoid degeneration, probably caused by heavy smoking.

author, and the benign quality of the papilloma in Fig. 15 could be expected only from the young age of the subject and the fact that he never had smoked.

Not only benign lesions of the epithelium have to be taken into account in the differential diagnosis of squamous cell carcinoma and its precursors but also other malignancies such as sarcoma, lymphoma, and melanoma, even if these are much less frequent.

### CONCLUDING REMARKS

It should be clear that even very insignificant changes of the vocal fold epithelium should raise the suspicion in the endoscopist of possible malignancy and be treated accordingly.

One method during the clinical examination to rule out adhesion of the mucosa, as it may be caused from invasive growth of a lesion, is by laryngeal stroboscopy (9). However, this is an examination requiring favorable anatomical conditions and adequate cooperation from the patient as well as extensive experience from the examiner. The only secure way to rule out premalignancies and malignancies is by microscopic evaluation of adequate biopsies, and this is also the only method to establish a correct diagnosis.

The biopsies of small epithelial lesions on the vocal fold mucosa should be made as local excisions from the beginning, which at the same time represents an adequate treatment in many cases. The biopsy should be made by endolaryngeal microsurgery (microlaryngoscopy) as described by Kleinsasser (10), which also is an adequate method to search for changes of the laryngeal epithelium in the area of the lesion under focus. The microlaryngoscopic examination may be completed by endoscopy with a narrow, angled (preferrably 70°) optic to visualize the subglottic areas of the larynx and the lateral aspects of the laryngeal ventricles.

As was illustrated by the case shown in Figures 5 and 6, recurrences are not unusual, nor is the development of originally low-grade dysplasia into invasive carcinoma. Therefore, an organization of regular long-term follow-up examinations is peremptory in the here described cases.

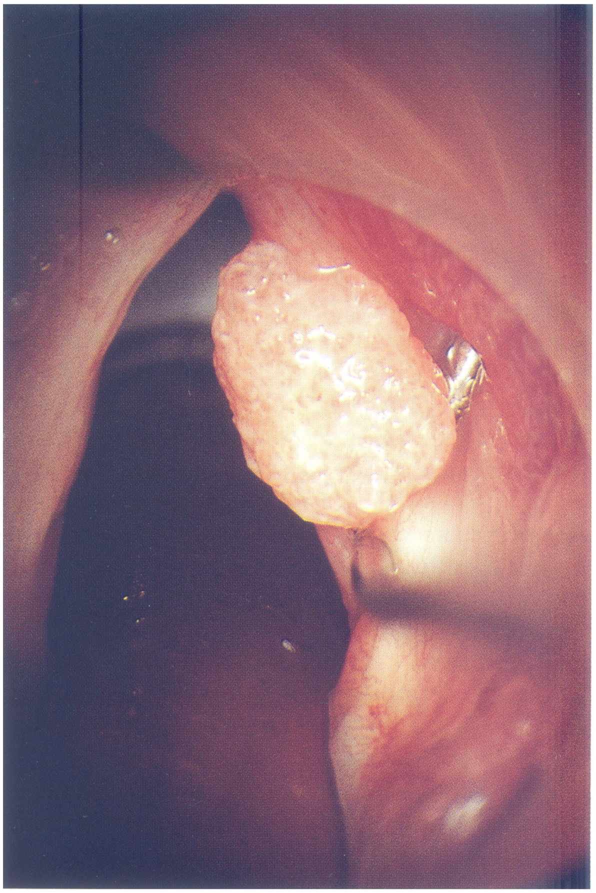

**Figure 15** Granulomatous benign papilloma of right vocal fold; man, 18 years. Notice practically identical appearance as invasive carcinoma in Fig. 9.

### REFERENCES

1. Hallén O. (ed., Larynxcancer, diagnostik och behandling. Nationellt vårdprogram. Onkologiskt centrum i Västsvenska sjukvårdsregionen, Göteborg/Kungälv, 1986.
2. Waterhouse JAH. Epidemiology of neoplasms of the larynx. In: Ferlito A (ed.), Neoplasms of the Larynx. Churchill Livingstone, 1993.
3. Kleinsasser O. Mikrolaryngoskopie und endolaryngeale Mikrochirurgie, Teil II. Rückblick auf 2 500 Fälle. HNO, 1974;22:69–83.

3a. Lehmann W, Pidoux J-M, Widmann J-J. Larynx Microlaryngoscopy and Histopathology. Inpharzam Medical Publications 1981.

4. Henry RC. The transformation of laryngeal leucoplacia to cancer. Laryngol Otology 1979;93:447–459.
5. Hellquist H, Olofsson J, Gröntoft O. Carcinoma in situ and severe dysplasia of the vocal cords. Acta Otolaryngol 1981;92:543–555.
6. Kleinsasser O. Über die Histogenese und das Wachstum junger Kehlkopfkrebse Z. Lar Rhin Otol, 1963b;42:499–520.
7. Kleinsasser O. Die Klassifikation und Differentialdiagnose der Epithelhyperplasien der Kehlkopfschleimhaut auf Grund histomorphologischer Merkmale. II. Mitteilung. Z. Lar Rhin Otol 1963a;42:339–362.
8. Friedman I, Ferlito A. Precursors of squamous cell carcinoma. In: Ferlito A (ed.): Neoplasms of the Larynx. Churchill Livingstone 1993.
9. Kitzing P. Stroboscopy—a pertinent laryngological examination. J Otolaryngol 1985;14:151–157.
10. Kleinsasser O. Mikrolaryngoskopie und endolaryngeale Mikrochirurgie. Technik und klinische Befunde FK, Schattauer 1968.

